# Understanding public discourse surrounding the impact of bitcoin on the environment in social media

**DOI:** 10.1007/s10708-023-10856-z

**Published:** 2023-03-27

**Authors:** Sachith Mankala, Audhav Durai, Anvi Padiyar, Olga Gkountouna, Ron Mahabir

**Affiliations:** 1 Fairfax High School, Fairfax, VA 22030 USA; 2 Thomas Jefferson High School for Science and Technology, Alexandria, VA 22312 USA; 3 Department of Computer Science, University of Maryland, College Park, MD 20742 USA; 4 Geographic Data Science Lab, Department of Geography and Planning, University of Liverpool, Liverpool, L69 3BX UK

**Keywords:** Bitcoin, Environment, Social media, Topic modelling, Public opinion

## Abstract

Increasing public concerns about the environment have led to many studies that have explored current issues and approaches towards its protection. Much less studied, however, is topic of public opinion surrounding the impact that cryptocurrencies are having on the environment. The cryptocurrency market, in particular, bitcoin, currently rivals other top well-known assets such as precious metals and exchanged traded funds in market value, and its growing. This work examines public opinion expressed about the environmental impacts of bitcoin derived from Twitter feeds. Three primary research questions were addressed in this work related to topics of public interest, their location, and people and places involved. Our findings show that factions of of the public are interest in protecting the environment, with topics that resonate mainly related to energy. This discourse was also taking place at few similar locations with a mix of different people and places of interest.

## Introduction

The last decade has witnessed unprecedented growth and advancement in technology. One major upward growth trend is that of cryptocurrency, the most well-known of which is bitcoin. Bitcoin was introduced in 2008 by anonymous entity, Satoshi Nakamoto, in wake of the global financial crisis that was occurring at the time (Chapron, 2017). Compared to traditional payment systems, bitcoin is neither backed by any government nor controlled by a central bank. It uses a decentralized ledger that is distributed across a peer-to-peer network of users to facilitate electronic transactions among them (Badea & Mungiu-Pupăzan, 2021). As such, there is no need for a third party financial institution, and associated mediation costs, compared to typical e-commerce transactions on the internet (Nakamoto, 2008). These properties of bitcoin result in several advantages over traditional cash flow systems, including, better security, transparency, and trust in transactions between users (Underwood, 2016). As of November 2022, there were more than 190 million bitcoin users globally (Howarth, 2022), with bitcoin accounting for about 38% (more than doubling that of Ethereum, the second highest) of the global cryptocurrency market cap^1^ of USD $809 billion.

The economic value of bitcoin and other cryptocurrencies, and the novelty of its trading infrastructure has given rise to many studies. Broad thematic research areas include business and industry, internet of things, governance, data management, and privacy and security, among others (see Casino et al, 2019) for a more in-depth review). Additionally, studies have examined the link between bitcoin and the environment, with main focus on it’s relationship with clean energy and carbon allowances  (e.g., Dogan et al, 2022), the energy required for mining operations (e.g., Gallersdörfer et al, 2020; Küfeoğlu & Özkuran, 2019) and it’s link to carbon emissions (e.g., Mora et al, 2018; Stoll et al. 2019; de Vries et al, 2022). Few studies, such as De Vries and Stoll (2021), have also examined direct impacts to the environment, such as resulting e-waste (i.e., waste produced by discarding electrical or electronic equipment from hardware used in mining operations).

Bitcoin has also been studied via the lens of social media, providing a wealth information from millions of users globally on a range of different topics surrounding the cryptocurrency. These include work assessing it’s impact on the performance of bitcoin (e.g., Mai et al, 2018; Guégan & Renault, 2021) and its relationship to the COVID-19 pandemic  (e.g., Béjaoui et al, 2021), and changes in discussions (e.g., Burnie & Yilmaz, 2019) and the volume of activity of Twitter and Google searches (e.g., Mittal et al, 2019) with price changes. Other work have also investigated the education levels of users (e.g., Narman et al, 2018) and the evolution of topics using data on Reddit forums (e.g., Linton et al, 2017). In addition to Twitter and Reddit, other social media data have also been in debut. These include studies using data from platforms such as Telegram (e.g., Mirtaheri et al, 2021) and Discord (e.g., Nizzoli et al, 2020) to identify currency manipulation on social media platforms, and work by Huang et al. (2021), using data captured from the Chinese social media platform, Weibo, to predict price changes in bitcoin. While such studies highlight an existing relationship between public discussions on online social media platforms and crytocurrencies such as bitcoin, the topic of public opinion is often muted in such work.

Public opinion plays a critical role in informing policy, with topics that resonate longer with the public generally receiving more attention from decision makers (Burstein, 2003; Wlezien & Soroka, 2007). This is particularly true with major policies, and when political elites, political organizations, and salient issues are involved (Deborah, 2001; Woods, 2008; Agnone, 2007; Benegal, 2018). Further, the opinion from key persons in the public have also been found to have an impact on shaping the success of technology. For example, statements made by business magnate and investor Elon Musk about Dogecoin cryptocurrency in 2021, led to a price increase of 43% two hours following his statement (Oosterbaan, 2021). A similar *knock on effect* was reported by Huynh (2021), who analyzed bitcoin statements made on Twitter by former US President Donald Trump. That study found that tweets with negative sentiments could be used to predict returns, trading volumes, realized volatility, and jumps in Bitcoin. Understanding public opinion on key issues, such as the impact of bitcoin on the environment, therefore, represents an important task for understanding the communication loop that exists between the public and policy makers. In this two-way loop, topics that are important to the public should be reviewed and assessed by policy makers in order to understand the salience of these topics, why they are of concern/interest to the public, and, ultimately, to have them addressed in a timely manner. Policy makers also enact laws that affect the attitudes of people, which can ultimately shape public opinion. Such knowledge can be used as a performance measure to assess to what extent developed policies to address the impact of bitcoin on the environment are working. To the authors knowledge, this is the first study to address this issue, using social media data as conduit to study public perception surrounding the impact of bitcoin on the environment. This work further addresses the issue of the low signal-to-noise ratio from unrelated social media posts collected from online platforms such as Twitter (Saleem et al., 2014). Failure to deal with such issues can limit the efficiency and effectiveness of such data for understanding public opinion (Sherchan et al., 2017), and lead to misleading results (Myslín et al., 2013).

The rest of this paper is structured as follows. In Sect. 2, background information on public opinion is provided, with main focus on the environment, and how social media has been used. Section 3 presents the data and methodology used in this research. Section 4 presents the results and analysis, following which, in Sect. 5, a discussion is provided with remarks on future work.

## Background

There has been growing public concern about the environment and interest in finding sustainable ways to protect it (Pew Research Center, 2020; Egan et al., 2022). This has been in part due to increased observations about the environment, and resulting fear that humans are overusing the Earth’s natural resources. Santiago Fink (2016) suggests that humans may already be depleting these resources at a rate of 40% higher than the Earth’s natural replenish rate. Such concerns has manifested itself as an array of different studies that try to understand peoples’ perception of the environment, and their topical interests. However, this issue is multifaceted in that a person’s perception of an issue can be influenced by many factors, such as their surroundings and their specific interactions with the environment (Fowler, 2016). Nonetheless, previous work has delved into areas including climate change (e.g., Brulle et al, 2012), renewable energy (e.g., Qazi et al, 2019), air pollution (e.g., Bickerstaff & Walker, 2001), deforestation, and waste management (e.g., Cheng & Urpelainen, 2015), just to name a few.

There has also been a notable increase in data collected from additional sources about the environment; these have provided new opportunities to study public opinion. Traditionally, studies have mainly relied on the use of surveys to gather relevant information. Such work include survey data collected from large third-party institutions such as the Gallup organization (e.g., Reinhart, 2018; Egan et al, 2022), the National Opinion Research Center (e.g., Dunlap, 1991), and smaller focus groups that may be interviewed by researchers themselves (e.g., Shackley et al, 2004). Some researchers have also used telephone interviews to conduct surveys, such as Bloodhart et al. (2015), who conducted interviews with 2,000 Virginia residents to examine the influence of local TV weather information on climate change perceptions. Others, such as Matthews (2015) and Wiest et al. (2015), have reviewed blogs on climate change, while Feldman et al. (2011), using nationally representative survey data, have investigated the role that late satirical TV shows play on informing opinion about climate.

However, survey polls have several drawbacks as it relates to the data on public opinion being collected. One such issue is that they typically allow for a very narrow view of the topic that the specific poll is addressing, and miss the equally important larger social context surrounding the topic (Lin et al., 2013). They also fail to adequately capture complex issues that extend beyond a single discipline or stakeholder group (Chen & Tomblin, 2021). In addition, there has been increasing non-response rates (Groves & Peytcheva, 2008) and incidents of over-reporting (Hadaway et al., 1993), which may lead to biased or misleading results. Added to this, participants not interested in the survey may provide poor feedback that may also impact the quality of the results (Hargittai & Karaoglu, 2018). Further, survey polls represent the public’s view at a particular point in time, whereas the general nature of public opinion is fluid, with dynamic shifts and permeability of opinion overtime (Zhang et al., 2022), and often reactive to unfolding world events. Such issues, taken together, have led to the exploration of other approaches towards gathering information on public opinion.

More recently, with the advent of big data and related computing technologies, social media data has emerged as a valuable source of data on public opinion about the environment. One prominent such source is Twitter, an online micro blogging platform with close to 400 million users (Shepard, 2023). The share magnitude of the number of users, the ability to share and exchange public views on a range of different technology affordances (e.g., laptops, tablets, and mobile phones), along with it’s global reach, makes platforms such as Twitter a rich source of information on public opinion.

Reyes-Menendez et al. (2018), for example, used this data and a topic-based sentiment analysis approach to study public sentiments related to sustainable care of the environment. A similar approach was used by Zhang et al. (2022) to identify perceptions associated with green house gas emissions and renewable energy. Further work has also explored the spatial and temporal patterns in tweeting activity relating to climate change (e.g., Kirilenko & Stepchenkova, 2014; Kirilenko et al, 2015), with additional studies establishing links between climate related events and people’s sentiments (e.g., An et al, 2014), and identifying different triggers that cause changes in the public’s emotional state (e.g., Cody et al, 2015). Studies have also used topic modelling to understand public opinion surrounding air pollution (e.g., Tvinnereim et al, 2017) and climate change policy (e.g., Wei et al, 2021). Moreover, other work have examined the nexus between human poverty and it’s impact on the environment (e.g., Cheng & Urpelainen, 2015); however, to date, there has been little investigation on the topic of how the cryptocurrency industry is impacting the environment.

The growth of the cryptocurrency industry over the last decade has transformed the way in which the digital economy has typically operated. This is owing in large part to the underlying technology on which most cryptocurrencies are built, that is, blockchain^2^. Blockchain was created by Haber and Stornetta (1990) as a decentralized approach for recording transactions and keeping track of assets. It was most notably popularized by bitcoin, a crytocurrency that uses blockchain to conduct cash transactions on the web. However, while it continues to fill current gaps in traditional banking systems through its peer-to-peer system (DeVries, 2016), the amount of energy required for bitcoin operations has been a double-edged sword. A recent report, for example, suggested that bitcoin consumes 121.36 terawatt-hours (TWh) of energy a year, which is more than the country consumption of Argentina (121 TWh), the Netherlands (108.8 TWh) and the United Arab Emirates (113.20 TWh), and is gradually on its way to surpass Norway (122.20 TWh) (Criddle, 2021). This high energy requirement accounts for about 1% of global consumption (Nahar, 2022), and leads to about 36.95 megatons of CO_2_ being produced from mining operations, which is comparable to emissions from the country of New Zealand (Browne, 2021). Further, the result of cooling operations for bitcoin mining companies that use natural water bodies have shown elevated water temperatures, affecting local communities and aquatic ecosystems at those locations (Hamacher, 2021).

The large amount of energy required by bitcoin is mainly due to the approach its uses to ensure that all legitimate transactions are recorded on the blockchain and that each copy of the blockchain contains all valid transactions. Bitcoin, in particular, use a *consensus* algorithm call Proof-of-Work (PoW), which requires computers involved in transactions (also known as miners) to prove their *work*. To do so, computers must solve a mathematical problem, with the fastest computer to do so gaining a reward. Since many computers want the reward, a lot of energy is used in this competitive process (Mingxiao et al., 2017). An alternative consensus mechanism, Proof-of-Stake (PoS), was developed as a low-cost, low-energy consuming alternative to the PoW algorithm. In PoS, computers involved in transactions (also know as validators) are chosen to validate transactions. To become a validator computers must deposit a specific amount of coins into the network (i.e., their *stake*), with nodes with higher stakes having a higher random chance of being choosen as a validator. If the validator approves a valid transaction, they receive the reward associated with the transaction. However, if they approve a fraudulent transaction, they loose a part of their stake, thus penalizing fradulent validators and requiring much fewer computers to achieve consensus on the blockchain network (Mingxiao et al., 2017). While many other more recent cryptocurrencies, such as Cardano, Solana, Avalanche, Tron, Cosmos, EOS, Tezos, and Etherium, use PoS, as discussed in Sect. 1, bitcoin still remains the most widely used crytocurrency in today’s market. As such, its usage remains a big issue for the environment.

Veering to the academic community, bitcoin has also been the subject of much heated debate, with studies that analyze the good and bad aspects about its usage, and future outlook (Rahardja et al., 2021). There however remains a gap in our knowledge about public opinion surrounding bitcoin, especially as it relates its direct impacts to the environment. Recent work by Badea and Mungiu-Pupăzan (2021), for example, examining scientific articles on bitcoin between 2016 to 2020 found only 12 articles relating to environmental impacts. Of those articles, most were focused on the energy usage of mining operations, providing a very narrow view of the environment. The public’s perception surrounding a technology plays an important role in its successful adoption (Upham et al., 2015; Johnson & Tyson, 2020), and therefore, failure to take this view into account may limit our understanding on the true potential of bitcoin and its use as a sustainable technology. Further, public opinion represents one part of a larger news awareness ecosystem (Mahabir et al., 2018) that continues to shape and be influenced by other components of this system, such as public policy and the news. Thus, a study of this nature is necessary if we are to advance our understanding of the role that public opinion plays in such systems, and their physical manifestation on the environment. This study helps address this gap in research by analyzing social media data to understand public opinion surrounding the impacts of bitcoin on the environment. Towards this goal, we address three main questions along this line of inquiry. First, what are there specific topics that are of public interest (i.e., the what)? Second, where are these discussions taking place (i.e., the where)? Finally, who are the prominent entities of interest to the public when it comes to discourse surrounding the environmental impacts of bitcoin (i.e., the who)?

## Methods

**Fig. 1:**
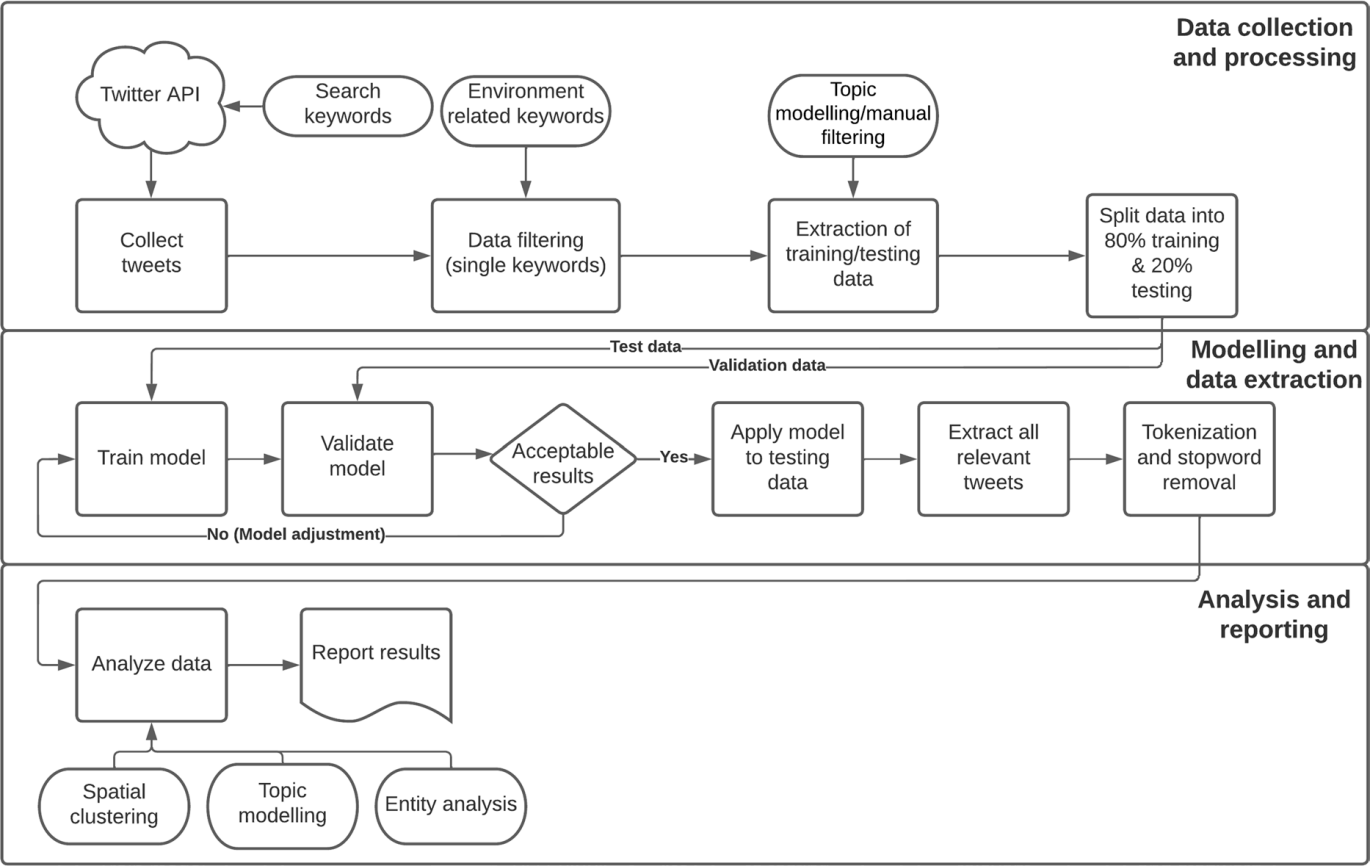
Overview of methodology

The methodology is divided into three steps: data collection and pre-processing, modelling and data extraction, and analysis and reporting. These steps are shown in Fig. 1 and discussed in greater detail in the subsections that follow.

### Data collection and pre-processing

The social media data used in this study were collected via the Twitter platform using their free Application Programming Interface (API) service for the period June 1, 2018 to July 31, 2020. While the start date remains the arbitrary point in time that we began collecting the data, the stop date was influenced by a significant decline in news articles (via the online Google news portal) discussing bitcoin and the environment from this point on. Following this time, most articles primarily focused on topics relating to the COVID pandemic. There were a few spikes in related articles after the stop date, however, the API service only allowed a search archive window of 7 days (Twitter, 2022). As a result, this made any new data collection after the stop date difficult to seamlessly integrate with our primary data collection period, that is, significant periods of no data would occur. Nevertheless, for the research questions that are being addressed in this study, the specific data period was determined to be adequate.

As discussed in Sects. 1 and 2, Twitter has been widely used for studying bitcoin and public opinion surrounding a range of different topics. Our choice Twitter, as compared to other sources of social media data, was informed by this, as well as other aspects. Another reason was accessibility of the data. Compared to other platforms such as Facebook, Twitter data is much more accessible via its API. Also, while sources such as Reddit forums provide more focused conversations (i.e., much lower amounts of noise), compared to Twitter, as previous research has shown (e.g., Priya et al., 2019), there tends to be much more fluid activity on Twitter, making this is good source of data when studying temporal activity, which is investigated in this research. Further, Twitter provides location information on users, which can be used to identify users located in the US, our study area. Platforms such as Reddit, on the other hand, do not provide this information, making it difficult to filter activity to a specific location. Moreover, while other social media sites such as YouTube and Instagram are also accessed by millions of people across the US daily, the exchange of information follows a much more niche group of users. For example, YouTube is typically used to post/reply to comments about videos, while Instagram is used primarily for sharing pictures. Twitter, on the other hand, has been developed with the principal directive of sharing public viewpoints through short bursts of text, and with the opportunity to embed other multimedia content such as images. In addition, the share magnitude of research on Twitter, and developed algorithms made openly available of platforms such as GitHub to collect and mine this data for analysis, makes Twitter a suitable option for this research.

The search terms, *bitcoin* and *cryptocurrency*, were first used to collect data from the API. These terms were broad in scope and used to capture as much information related to bitcoin as possible in the data provided by the API, which would then be further filtered as discussed next. According to Yuan et al. (2021), using more specific keywords to gather information may also limit the scope of understanding a particular topic, and the geographic locations involved. Only tweets with latitude and longitude information were retained, and this information then used to filter the data to the continental US. Further, only tweets in English were used in this research. These were identified using the language option provided by the API. This decision to use English tweets was driven by two main factors. First, the most spoken language in US households is English, with 241 million (followed by Spanish with 42 million) (Hernandez & Dietrich, 2022). Second, most natural language models used today perform adequately with English texts but not so well for other languages (Tsarfaty et al., 2020).

Following the above filtering of the data, at this point, the data still contained a lot of noise as a result of the broad search terms. To further filter relevant tweets, a survey of the literature was undertaken to identify keywords that were typical of articles published about the environment, along with articles specific to bitcoin and the environment. Sources for scientific articles included the Web of Science and Google Scholar. Table 1 shows the most common terms about the environment. Only tweets that contained at least one of these terms were retained. This resulted in 130,000 tweets for the US.

**Table 1:** Environment keywords

Air contamination	Earth	Global warming	Power
Air pollution	Electrical	Green	Renewable
Atmosphere	Electricity	Greenhouse gas	Sustainable
Coal	E-waste	Greenhouse effect	Twh
Carbon dioxide	Electronic waste	Hydroelectricity	Terawatt-hour
Carbon footprint	Energy	Hydropower	Water contamination
Climate	Energy-saving	Nature	Water pollution
Climate change	Emission	Natural resource	Weather
Consumption	Environment	Planet	Wildlife
Contamination	Fossil fuel	Pollution	

After the above filtering step, the data still contained noise from irrelevant tweets. For example, some tweets discussed increased electricity usage without having any reference to the environment. Similarly, tweets with the term *green* sometimes referred to money and not the environment. Such occurrences are a familiar problem with social media data. One particular study by Lanyi et al. (2021), for example, found only 33% of tweets to be applicable following similar processing steps. In order to further filter tweets, 12,000 relevant and 12,000 non-relevant tweets were manually labelled by the authors. These were to be used to train a machine learning model (discussed further in Sect. 3.2) to classify the remaining tweets as relevant and non-relevant. Each relevant and non-relevant labelled corpus were split into 80% training data and 20% validation data. All remaining tweets were then kept as testing data.

### Modelling and data extraction

This step involves building a machine learning model to classify tweets using the training data. Specifically, a support vector machine (SVM) classifier was used, which previous studies that used text data have shown to yield good classification results (e.g., Lamb et al, 2013; Dilrukshi et al, 2013). Following the model training phase, the model was then evaluated with the remaining validation data. Further adjustments were made to model parameters to address low validation accuracy, and this process repeated until an acceptable level of accuracy was achieved. The final model achieved an overall classification accuracy of 88.5%, and was then applied to the test data to identify relevant tweets. Previous work by Kharde et al. (2016), comparing different approaches for opinion mining, reported overall classification accuracies between 74% (Lexical-based approach) to 86% (SVM-based approach). The one exception was an entropy weighted genetic algorithm approach developed by Abbasi et al. (2008) that achieved a classification accuracy of 91.7%. However, that study used features extracted from multiple languages, which was beyond the scope of the current study. Relevant tweets from the test data, together with relevant tweets from the training and validation data were then combined to create a new data corpus containing all relevant tweets. This resulted in 71,963 tweets, which were then tokenized, lemmatized, and cleaned of stopwords. Moreover, repeated words were removed from individual tweets.

### Analysis and reporting

The tokenized tweets were used in various types of analyzes. Specifically three types of analyzes that address our research questions were used. To extract and study topics of public interest, the Latent Dirichlet Allocation (LDA) algorithm was applied to all the tweets. LDA is an unsupervised machine learning statistical topic model. Utilizing a method of unsupervised classification of documents on a given corpus of data, it is able to extract hidden and relevant topics from the twitter corpus (Blei et al., 2003). For the LDA model, the Dynamic Topic Modelling (DTM) was then performed to study the monthly (28 days) evolution of topics (Blei & Lafferty, 2006). As both LDA and DTM can result in an arbitrary number of clusters, the topic coherence score was computed and used to determine the optimal number of topics (Röder et al., 2015).

To understand the spatial distribution of topics, tweets for each topic were identified and the density-based spatial clustering of applications of noise (DBSCAN) algorithm (Ester et al., 1996) applied to them. This algorithm works by grouping together points on a map that are geographically close and dense. Two parameters are required for DBSCAN: an epsilon value (i.e., distance) and the minimum number of points within a cluster. Epsilon was found by first using the Nearest Neighbor algorithm (Cover & Hart, 1967) to find the minimum distance to each points’ nearest neighbor. Next the *knee point* was computed following the work of Satopaa et al. (2011). This represents the optimal trade-off (local minima) between the highest density of points and the smallest distance between them. For the minimum number of points, a value of 100 was used. Too few number of points would lead to too many clusters, missing the broad community patterns in the data. Too large number of points could lead to over generalization of clusters. In addition, DBSCAN is very computationally intensive. The selected optimum value, therefore, took these factors into account, along with an examination of different epsilon values and their resulting spatial distribution of tweets.

Finally, to study the prominence of entities, the original data, prior to tokenization, was used. Individual tweets were parsed to the TextRazor Name Entity Recognition (NER) online tool (TextRazor, 2022) and entities relating to people, and places of interest were then identified for each tweet. Prominent entities were determined by selecting the most frequent entity in each category for all the data, for monthly intervals (i.e., 28 days). Following this, a record or all results were reported, as will be discussed further in the next section.

## Results

The results are presented in accordance with the three research questions that this work seeks to answer. These questions were: what were the topics of public interest (Sect. 4.1), where was this discourse taking place (Sect. 4.2), and who are the main entities that resonated with the public in these discussions (Sect. 4.3). The subsections that follow provide the results for each of these questions. However, before moving forward, it is also important to note, at this point, that because we use a sample of tweets, none of the results should be interpreted as being representative of the larger population. This is an ongoing challenge with socio-technological platforms such as Twitter and has been the subject of many prior studies (e.g., Olteanu et al, 2019; Malik et al, 2015). Results should therefore be interpreted as the opinions of a limited sample of people that use Twitter, and which may be expressive of some of the wider issues surrounding public perception of the impact of bitcoin on the environment.

### Topic saliency

**Fig. 2:**
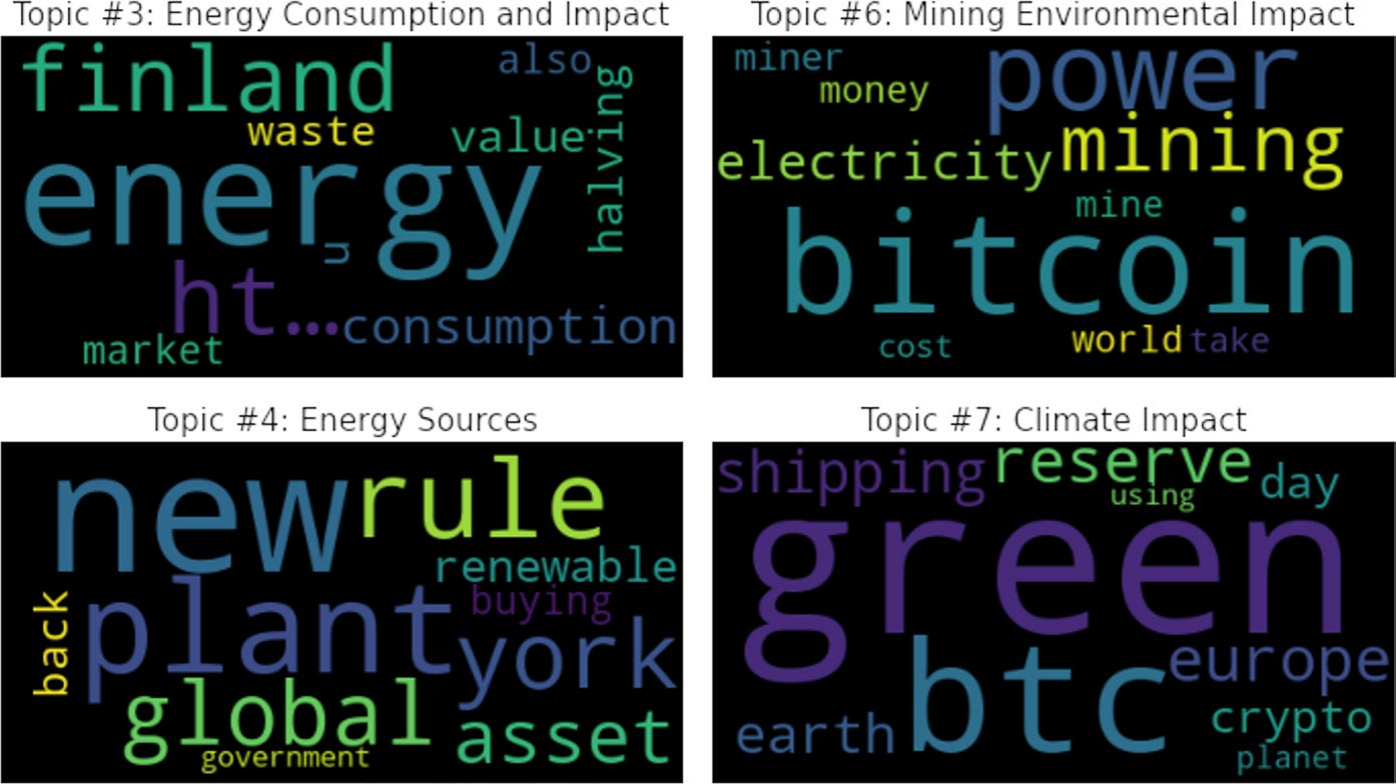
LDA topics

As it relates to topics of public interest, 8 main topics were found to be co-circulating. However, on further examination of these topics, only 4 (i.e., topics 3, 4, 6,and 7) were found to be relevant to the environment. Such results highlight key limitations with relying on search terms alone to identify relevant data on social media platforms such as Twitter. The 4 relevant topics are visualized as word clouds in Fig. 2. Topics 3, 4, and 6 are related to energy. In topic 3, there is interest in energy consumption from bitcoin operations and it’s wastage. Finland is mentioned in many tweets, drawing attention to several news articles, which reported that the energy used for bitcoin mining operations was almost on par with electricity usage for the entire country of Finland.

Topic 4 is mainly concerned with different sources of energy, including traditional power plants and some renewable sources (albiet a very small percentage of the global consumption). We also see mentions of “york” and “government”, referring to discussions surrounding investigation of bitcoin energy usage by government in the state of New York. Topic 6 is mainly related to the cost of energy used for bitcoin mining operations. Finally, in topic 7, people are interested in bitcoin being harmful to the green climate initiative and presenting long term risks to the planet. Overall, our sample of users and derived topics are suggestive of a vested interest/concern by the public in the energy usage of bitcoin, it’s impacts to the environment, and renewable energy as a way forward.

**Fig. 3:**
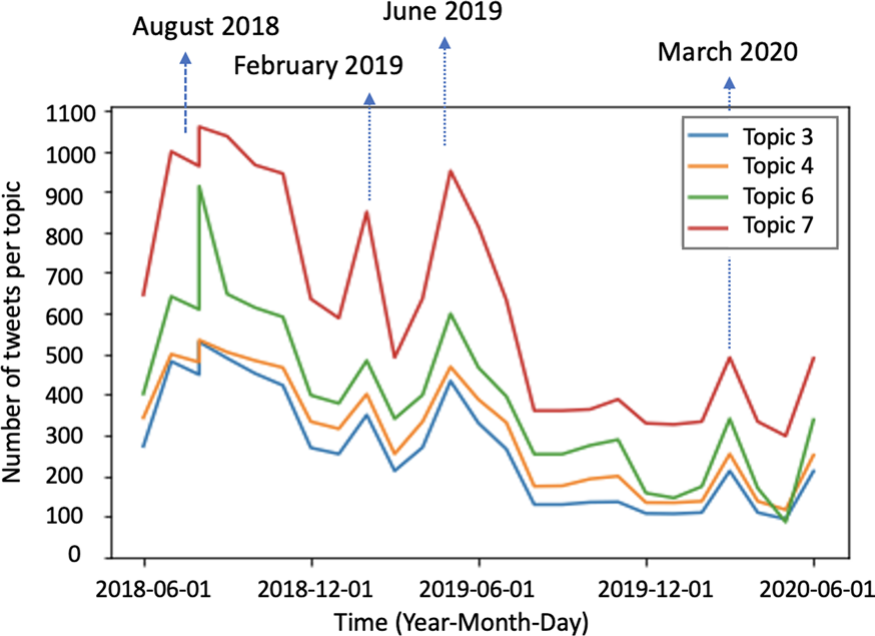
DTM topics over time

The results from DTM are shown in Fig. 3. Similar to the above, the same 4 topics (i.e., 3, 4, 6 and 7) re-emerged as being relevant. Figure 3 shows that these topics persist over time in the data. Also evident is that all time series follow the same general temporal pattern. Topics 3, 4, and 6, in particular, have very similar temporal patterns, which is reasonable given that they are more closely related. Topic 7, on the other hand, consistently has the most amount of tweets, and as discussed before, suggests that, at least for our sample of users, the public’s interest in protecting the environment.

Further taking a more contemporaneous view of the data, in Fig. 3, 4 main peaks can be observed in August 2018, February 2019, June 2019, and March 2020 respectively. For the first peak (i.e., August 2018), both topics 3 and 4 surface a lot of discussion surrounding the large amounts of energy used by bitcoin. Topic 6 discusses impacts to the green climate initiative, while topic 7 discussions revolve around sustainable energy sources, with large number of posts mentioning wind power as a viable option.

In February 2019 (i.e., second peak), topic 3 conversations mainly focused on sustainable blockchain technology as an approach to help offset energy costs. In particular, there is a lot of discussion following the unveiling of Apollo, and its associated protocol, Hermes, as a sustainable cryptocurrency replacement for bitcoin. Topics 3, 6, 7 have similar discussions as before, with topic 7 mainly focusing on the use of solar panels as a renewable energy alternative for meeting the energy requirements of mining operations.

The third peak in June 2019 continues to have discussions centered on energy utilization for topics 3 and 4 respectively. Las Vegas in particular is mentioned, with news articles highlighting recent research at the time, which found that the electricity required for bitcoin generates as much carbon dioxide as the city of Las Vegas (Stoll et al., 2019). Topic 6 discussions remain similar as before, while for topic 7, bitcoin mining in Iran is at the forefront of discussion. In particular, there is a lot of discussion surrounding Iranians setting up mining operations in mosques because of the free electricity that these institutions receive from their government.

Finally, for the last peak in March 2020, energy consumption continues to dominates discussion in topics 3 and 4. Topic 6 continues to see discussion revolve around protecting the environment and the green energy initiative. While in topic 7, New York and energy wastage is mentioned, owning a large part to investigation of bitcoin energy usage by the government of New York.

Similar to LDA, the DTM results suggest that their is interest in the environment and learning of more sustainable ways to co-exist with bitcoin operations. However, at the same time, the DTM results show that while topics do persist over time, they are dynamic, with some variation in discussions occurring from one point in time to another. This is important to note as very specific keywords used to collect the data used in this project may have missed some of these more subtle changes over time. Further, there is an association between expressed interest and information coming from news agencies. Previous work by McCombs et al. (2011) have shown a deep rooted and two-way link between news and public opinion, with each being able to influence the other. Others work further suggest that social media should be included, forming part of a much larger news awareness ecosystem (e.g., Wang et al, 2021; Mahabir et al., 2018). In the case of our study, while were are unable to conclusively say that the population at large is featured in such a system, we still acknowledge an association of this kind in our sample of users.

### Spatial distribution of topics

**Fig. 4:**
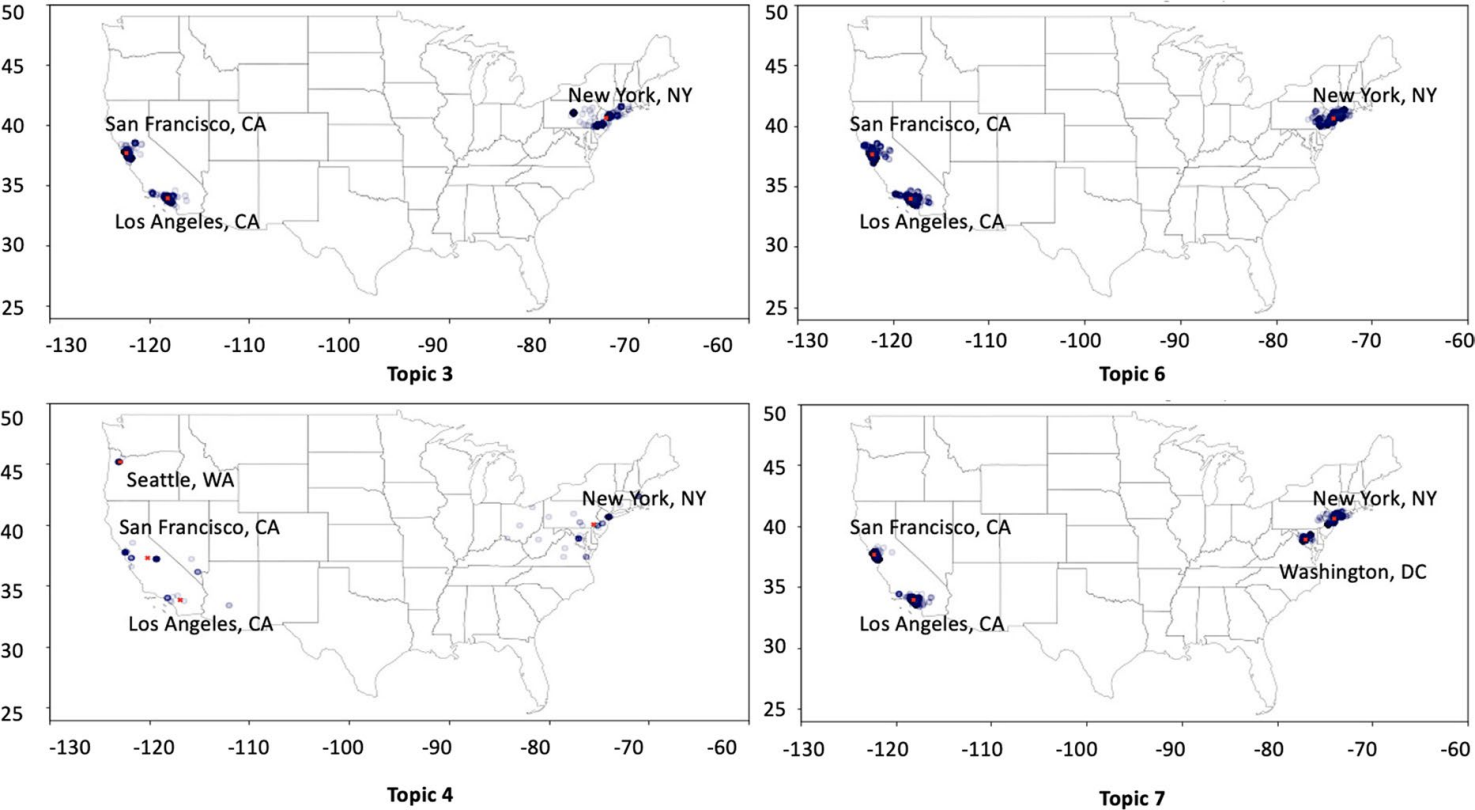
Spatial distribution of topics

Following the analysis of topics, individual topics were then plotted on a map of the continental US and further examined. Figure 4 shows the spatial distribution of each topic. Topic 3 resonates mainly around large cities along the eastern and western regions of the US: New York, San Francisco, and Los Angeles. Whereas the former location represents a large financial center, the latter two are very large technology centers that support bitcoin initiatives.

Topic 4 had similar results to topic 3, however, with a lot less density of tweets. Topic 4 also includes another technology center, Seattle, Washington. Topic 6 had clustering around similar cities to topic 3, with a much larger density of tweets. Finally, topic 7 had similar clustering around large cities as topic 6, but with a smaller clusters of tweets. In addition, topic 7 includes the main policy center of the US, that is, Washington DC.

From the above findings, a few general observations can be made concerning the spatial distribution of topics. First, most discourse occur in and around large cities in the eastern and western parts of the US. These cities represent major financial, technological, and policy hubs for bitcoin. Second, all topics (i.e., 3, 4, 6, and 7) show similar spatial distribution, albeit with different density of tweets, suggesting an association between them. As discussed in Sect. 4.1, these mainly center on energy usage for bitcoin operations. Finally, as well-known as bitcoin may be, and at least for the study period examined in this research, interest in the impact of bitcoin on the environment are concentrated in few locations only in the US. Accordingly, a recent survey by the Pew Research Center (Perrin, 2021) reported that 86% of Americans have at least heard about crytocurrencies like bitcoin, with 16% further stating that they have invested, traded or otherwise used at least one cryptocurrency. As such, while this particular work does not differentiate between Americans and non-Americans contributing to discourse within the US, the results may be related to a general lack of interest or lack of exposure of information surrounding the impact of bitcoin on the environment.

### Prominent entities

Turning to the prominent entities, Tables 2, 3, 4, 5 (see appendix) show the top 3 (based on frequency) identified person entities for topics 3, 4, 6, and 7 respectively. This table includes *NA* values, which were used to indicate instances where no person entity was identified by the state-of-the-art TextRazor NER platform. Each identified entity was manually verified through Twitter posts for the specific topic and period, and using the Google Search platform. All partially identified entities from TextRazor were fully identified using this approach. Through manual verification of samples, some entities were found to not be identified. This is not uncommon when working with user generated content, and is especially challenging with tweets due to the reduced amount of contextual information in these short messages (Derczynski et al., 2015). Nonetheless, several general observations can be made from the results in Appendix. First, person entities can be grouped into one of the following broad themes: finance and investment (e.g. venture capitalists), business (e.g., CEO of large technology corporations), politicians (e.g., congress personnel), news personal (e.g., TV anchors and reporters), computer scientists (e.g., the co-founder of the Etherium platform and Satoshi Nakamoto, the creator of bitcoin), academics (e.g., University professors that do research on bitcoin), and onscreen personel (e.g., actors, musicians and professional athletes). These results suggests that a broad range of persons are being sensitized, and are contributing to discussions surrounding bitcoin and the environment. Also, there is no specific patterns of types of entities per topic, but rather a mix of all entity types across topics. Second, the the frequency of prominent entities is low. This is especially the case for topic 4, suggesting that while there is interest in the environment, this level of interest is not as prominent as other societal issues such as climate change or poverty. The was in part shown in Sect. 4.2, where discussions were mainly centered within a few states and cities in the US. Finally, overtime, within and across topics, several entities were found to be more prominent than others, that is, they keep reappearing in discussions. These include Satoshi Nakamoto (the creator of bitcoin), Jeff Bezos (the CEO of Amazon), Donald Trump (former US President during the period for which the data was collected), and Elon Musk (business magnate and investor), among others. These entities play a critical role in (re)shaping public opinion about the impact of bitcoin on the environment.

Tables 6 and 7 (see appendix) show the results for identified place entities. In order for places to be compared within and across topics, places were aggregated to the country level. For example, those tweets that provided more granular location information, such New York city, when processed were replaced with the United States. Appendix shows a similar occurrence with *NA* values. As with the person entities, several general observations can be made with the place entities. First, while many countries are the topic of discussion globally, the United States and China occur the most in discussions. However, at least with respect to the United States, because only geolocated tweets within this country were considered, this may have in part helped inflate the frequency of this place entity. Second, compared to the person entities, with the exception of topic 4, the frequency of places is much higher, suggesting that people are especially interested in countries involvement and what they are doing to address the issue. Third, most of the discussion is centered on developed countries compared to low and middle income countries, which may be symptomatic of the former locations having the technological, financial and policy means to invest in bitcoin. Finally, possibly due to the close relationship between topics, countries tend to not be specific to any particular topic. Rather, they are salient within and across topics over time.

## Discussion

Increasing concerns about the environment over the last few decades have a prompted public interest on a wide range of environmental issues. These include air and water pollution, ozone depletion, forest degradation, loss of biodiversity, and ocean acidification, among others. Such attention has been in part influenced by the presence of social media as a medium connecting people to events and discussions taking place about the environment worldwide. This has allowed more people to contribute to these discussions, independent of background or expertise, and leading to greater diversity in topics of public interest (Conway et al., 2015; Lee et al., 2018). Sociotechnical systems with large followings, in particular, Twitter, are increasingly being used to help conceptualize and understand such human social behaviour, providing new insights on their evolution over time (Ferrara, 2020). A recent topic of public interest, in this respect, has been the impact of cryptocurrencies such as bitcoin on the environment. Most research to date have focused on the large amount of energy being used for mining operations to generate these digital currencies. Yet, little is still known about how public opinion is being shaped by discourse surrounding this issue on social media. This paper has explored this issue, using a sample of people from the Twitter platform, and addressing three lines of enquiry as it relates to the most popular cryptocurrency, bitcoin. Specifically our work answered the following research questions: What are the topics of public interest? Where are these discussions taking place? Who are the prominent entities of interest to the public when it comes to discussions surrounding the environmental impacts of bitcoin?

As it relates to our first research question, four topics were found to be co-circulating during the period for which data were collected, all related to energy. These were interest in energy consumption from mining operations, different sources of energy including renewable forms, the cost of energy, and how bitcoin is impacting green climate initiatives. Topics were also found to be persistent over time, following similar temporal patterns, but with different volumes of activity and in some cases, having small variation in topic from one point in time to another. With respect to the ebb and flow of public opinion, studies have shown public interest to be dynamic (e.g., Sha et al, 2020; Sasaki et al, 2014), and in part shaped by multiple aspects, including, topic salience (e.g., Burstein, Burstein (2003)), prior knowledge (e.g., Nowak et al, 1990), and reporting in the news (e.g., Page et al, 1987). In the current study, as will be discussed following, opinions expressed in the cohort of users showed that news agencies, along with various other actors may be playing a key role on shaping public opinion surrounding the impact of bitcoin on the environment.

With respect to our second research question, each of the four aforementioned topics were found to have similar geographic patterns within and around large cities along the eastern and western regions of the US. These locations represent major financial, technological, and policy hubs, and as it relates to bitcoin, each plays a pivotal role in it’s continued existence and success. For example, financial centers such as New York, is home to large stock market exchanges and brokerage firms, which allow investments in and the tracking of bitcoin assets. Major technological centers, such as San Francisco, contain a large number of companies that are continuously working to support the cyber-infrastructure of crytocurrencies, and developing new ways to make bitcoin a more sustainable technology. Finally, as it relates to policy centers like Washington DC, since bitcoin is a recent currency, in order to be successful, existing policies must be revised or new policies developed to incorporate bitcoin into existing financial systems. These three particular tenants, that is, finance, technology, and policy, were previously discussed in work by Ehrentraud et al. (2020) as part of a proposed conceptual framework to characterise fintech environments like bitcoin. Our results therefore show that this model can also be extended to understand public discourse online about such environments. However, only few such locations were leading discussions, and while a great majority of Americans are familiar with bitcoin, there is limited interest beyond these leading cryptocurrency centers. Recent work by Dai and Chen (2022) on the geography of the fintech industry in China found two major reasons for the uneven nature of fintech distribution: (1) they tend to be located in cities that have accumulated technologies in the fields of finance, e-commerce, data sciences, and security, and (2) there exists a positive relationship between the development of the fintech industry and the demand for fintech services. The two factors could similarly be at play when it comes to the fintech industry in the US.

Finally, our last research question examined the prominent entities being discussed in discourse about the environment. Different broad groups of people resulted from this analysis; those involved in finance and investment, business, politics, news reporting, computer science and related technology development, academics, and onscreen personnel. Some of these entities, in particular, well-known politicians and business magnates, were much more talked about compared to other remaining entities, suggesting that they play a pivotal role in shaping public opinion. These results are in line with the findings of recent work that found similar favorable influence from well-known persons (e.g., Jackson, 2018; Nownes, 2021). Several news articles about the environment were also found to be circulating within user posts on Twitter, which may in part be helping to set an agenda thereof, a topic that has been thoroughly studied for the last 40 years (McCombs & Shaw, 1993). Similar finding for social media were reported by Asur et al. (2011), noting that social media mainly acted as a vessel amplifying news content.

Further, as it relates to location entities, most discussions were about the US and China. However, many other countries were discussed through the discourse across topics with no particular pattern observed. With respect the US, the salience of this location is expected to be in part influenced by the US being our specific study area. Nonetheless, analysis of tweets that contained US entities show large discourse on the technical advancements in cryptocurency and development of more sustainable technology. As for China, the data showed a large amount of discussions about the large amounts of energy used for mining operations in in that country. This was highlighted by various news articles embedded within Twitter posts at the time showing China to be leading bitcoin mining operations.

The results of this study presents several noteworthy findings that can be used for informing public policy. First, it shows that while the public is interested in protecting the environment, few topics resonate with them as it relates to the impact of bitcoin. Policy makers can use this knowledge to help prioritize these concerns, in order to address them in a timely manner. This would also mean that for those topics that are of public interest but do not realize themselves in public spaces such as social media, they should further try to understand why this is the case, in order to foster better communication with the public. Second, there is a direct link between traditional news, key public figures and public opinion, which should be taken into account in matters of public policy. To ignore anyone of these actors when making decisions that impact the public could have adverse impacts. Finally, public opinion is dynamic and needs to be continually monitored and analyzed over time. Thus approaches such as the one used in this research to understand public opinion should therefore be continually used and improved to ensure that the people that make decisions and those that are impact understand each other.

It is also important to note that there were several limitations with this work, which provide opportunities for future research. One such limitation was that the keywords used and collected tweets were in English. As such, the results can only be interpreted with respect to the English speaking population and not the wider population of the US. While 241 million people speak English at home in the US, 42 million are Spanish speaking (Hernandez & Dietrich, 2022), which when analyzed could provide additional important insights into public opinion across the US. Further work should therefore consider other languages, in addition to using other important keywords. Bots were also not distinguished from human posts, which could lead to inflated activity in some cases. Next, as Twitter is constantly updating their rules and policies, accounts that are suspended at the time the data was collected would not be included in the data. In addition, the data was collected using Twitter’s free API, which only gives a sample of the data. Future work should therefore utilize a more complete dataset, or at least determine that the sample data provided by Twitter is an adequate representation of the population and topic under investigation. Finally, data from other social media platforms should be investigated.

## Appendix

See Tables 2,3,4,5,6,7.

**Table 2:** Person entities—Topic 3

Date	TOPIC 3					
	First Entity	Freq	Second Entity	Freq	Third Entity	Freq
05/01/2018	Scott Melbye (President/CEO at Uranium Royalty Corp)	1.0	NA	NA	NA	NA
05/29/2018	Remi Vladuceanu (Social Media Strategist)	1.0	Vladimir Putin (Russian President)	1.0	Sergi de Cornudella (international interpreneur)	1
07/27/2018	Satoshi Nakamoto (developer of bitcoin)	1.0			NA	NA
08/24/2018	Yitzhak Rabin (assassination of Israeli Prime Minister)	3.0	William Shatner (Actor)	1.0	NA	NA
09/21/2018	Elon Musk (business magnate and investor)	2.0	Gary Gensler (Chairperson, U.S. Securities and Exchange Commission)	1.0	Brianna Wu (Financial Advisor)	1.0
10/19/2018	Larry Fink (CEO of BlackRock)	1.0	NA	NA	NA	NA
11/16/2018	Jack Ma (Chinese business magnate, investor and philanthropist)	3.0	Kelly O’Dwyer (former Australian politician)	1.0	Adam Davidson (American journalist)	1.0
12/14/2018	Vitalik Buterin (co-founder of Ethereum)	1.0	Tyler Winklevoss (American investor)	1.0	NA	NA
01/11/2019	Joe Rogan (American podcaster)	1.0	Gerald Butts (Canadian political consultant)	1.0	NA	NA
02/08/2019	Bernie Sanders (US politician)	2.0	American journalist	1.0	NA	NA
03/08/2019	Jerome Powell (Chair of the Federal Reserve of the United States)	1.0	Jack Ma (Chinese business magnate, investor and philanthropist)	1.0	NA	NA
04/05/2019	Stephen Miller (CEO of Crypto Current - Education platform)	1.0	Julian Assange (Australian editor)	1.0	Donald Trump (US President at the time)	1.0
05/03/2019	Donald Trump (US President at the time)	5.0	Jack Ma (Chinese business magnate, investor and philanthropist)	2.0	Jeffery Frankel (international macroeconomist)	1.0
05/31/2019	Svitlana Volkova (data scientist at Pacific Northwest Nationa)	1.0	Christian Stoll (Academic)	1.0	Omar Syed (Co-Founder Shardeum)	1.0
05/28/2019	Steve Wozniak (Apple co-founder)	1.0	Roger Ver (investor in Bitcoin)	1.0	Anthony Pompliano (entrepreneur and investor)	1.0
07/26/2019	Jeffrey Epstein (now deceased American financier)	1.0	NA	NA	NA	NA
08/23/2019	NA	NA	NA	NA	NA	NA
09/20/2019	Pat Geisinger (CEO of Intel)	1.0	Nancy Pelosi (US congresswoman)	1.0	Brad Garlinghouse (CEO of Ripple - provider of crypto solutions)	1.0
10/18/2019	Brad Garlinghouse (CEO of Ripple - provider of crypto solutions)	2.0	NA	NA	NA	NA
11/15/2019	Phil Zimmermann (American computer scientist and cryptographer)	1.0	David Pan (Crypto & Blockchain Companies Reporter)	1.0	NA	NA
12/13/2019	Milton Friedman (now deceased American economist)	1.0	NA	NA	NA	NA
01/10/2020	NA	NA	NA	NA	NA	NA
02/07/2020	Greta Thunberg (Swedish environmental activist)	1.0	Donald Trump (US President at the time)	1.0	NA	NA
03/06/2020	Brad Garlinghouse (CEO of Ripple - provider of crypto solutions)	1.0	NA	NA	NA	NA
04/03/2020	Sal Ternullo (Director and Co-lead of KPMG’s Cryptoasset Services)	1.0	NA	NA	NA	NA
05/01/2020	Elon Musk (business magnate and investor)	1.0	Donald Trump (US President at the time)	1.0	NA	NA
05/29/2020	NA	NA	NA	NA	NA	NA

**Table 3:** Person entities—Topic 4

Date	TOPIC					
	First Entity	Freq	Second Entity	Freq	Third Entity	Freq
05/01/2018	NA	NA	NA	NA	NA	NA
05/29/2018	NA	NA	NA	NA	NA	NA
07/27/2018	NA	NA	NA	NA	NA	NA
08/24/2018	NA	NA	NA	NA	NA	NA
09/21/2018	NA	NA	NA	NA	NA	NA
10/19/2018	Marin Katusa (professional investor)	1.0	NA	NA	NA	NA
11/16/2018	NA	NA	NA	NA	NA	NA
12/14/2018	NA	NA	NA	NA	NA	NA
01/11/2019	NA	NA	NA	NA	NA	NA
02/08/2019	NA	NA	NA	NA	NA	NA
03/08/2019	NA	NA	NA	NA	NA	NA
04/05/2019	NA	NA	NA	NA	NA	NA
05/03/2019	NA	NA	NA	NA	NA	NA
05/31/2019	NA	NA	NA	NA	NA	NA
05/28/2019	NA	NA	NA	NA	NA	NA
07/26/2019	NA	NA	NA	NA	NA	NA
08/23/2019	NA	NA	NA	NA	NA	NA
09/20/2019	NA	NA	NA	NA	NA	NA
10/18/2019	NA	NA	NA	NA	NA	NA
11/15/2019	NA	NA	NA	NA	NA	NA
12/13/2019	NA	NA	NA	NA	NA	NA
01/10/2020	NA	NA	NA	NA	NA	NA
02/07/2020	Peter McCormack (journalist/podcaster)	2.0	NA	NA	NA	NA
03/06/2020	NA	NA	NA	NA	NA	NA
04/03/2020	NA	NA	NA	NA	NA	NA
05/01/2020	NA	NA	NA	NA	NA	NA
05/29/2020	NA	NA	NA	NA	NA	NA

**Table 4:** Person entities—Topic 6

Date	TOPIC 6					
	First Entity	Freq	Second Entity	Freq	Third Entity	Freq
05/01/2018	William Shatner (Actor)	28.0	Trevor Griffiths (Analyst, Power Ledger)	1.0	Nichiren Dinzeo (Game developer)	1.0
05/29/2018	Steven Dong (CEO of EtainPower)	2.0	William Shatner (Actor)	1.0	NA	NA
07/27/2018	Thomas Uhm (Global Crypto Institutional Sales & Trading team)	1.0	Jon Truby (director of the Centre for Law and Development, Qatar U.)	1.0	Satoshi Nakamoto (developer of bitcoin)	1.0
08/24/2018	Satoshi Nakamoto (developer of bitcoin)	4.0	Toby Keith (Bitcoin trader)	1.0	Sergey Nazarov (co-founder of Chainlink)	1.0
09/21/2018	Anatoly Yakovenko (co-founder of the Solana project)	2.0	NA	NA	NA	NA
10/19/2018	Hal Finney (computer scientist)	8.0	Roger Ver (investor in Bitcoin)	1.0	NA	NA
11/16/2018	Satoshi Nakamoto (developer of bitcoin)	3.0	Satoshi Nakamoto (developer of bitcoin)	2.0	Michael Novogratz (American investor)	1.0
12/14/2018	Joachim Breitner (Computer scientist)	16.0	Jordan Peterson (Canadian clinical psychologist)	2.0	Satoshi Nakamoto (developer of bitcoin)	1.0
01/11/2019	Joachim Breitner (Computer scientist)	3.0	Donald Trump (US President at the time)	2.0	Vladimir Putin (Russian President)	1.0
02/08/2019	Bernie Sanders (US politician)	5.0	Satoshi Nakamoto (developer of bitcoin)	3.0	Steve Baker (British politician)	1.0
03/08/2019	Satoshi Nakamoto (developer of bitcoin)	2.0	Bernie Sanders (US politician)	2.0	Tucker Carlson (American reporter)	1.0
04/05/2019	Craig Steven Wright (Computer scientist)	3.0	Satoshi Nakamoto (developer of bitcoin)	2.0	Nipsey Hussle (deceased American rapper)	2.0
05/03/2019	Mati Greenspan (CEO & Founder, Quantum Economics)	4.0	Tim Draper (American venture capitalist)	3.0	Mike Pompeo (American politician)	3.0
05/31/2019	Christian Stoll (Academic)	2.0	Cameron Winklevoss (American cryptocurrency investor)	2.0	NA	NA
05/28/2019	Rajabi Mashhadi chairman & managing director of Iran Grid Management Company	10.0	Elon Musk (business magnate and investor)	2.0	Vitaly Dmitriyevich (co-founder of Ethereum)	1.0
07/26/2019	Donald Trump (US President at the time)	2.0	Satoshi Nakamoto (developer of bitcoin)	1.0	George Kamiya (Digital/Energy Analysis)	1.0
08/23/2019	Satoshi Nakamoto (developer of bitcoin)	1.0	Satoshi Nakamoto (developer of bitcoin)	1.0	Maria Jones (Finance Manager at Crypto.com)	1.0
09/20/2019	Satoshi Nakamoto (developer of bitcoin)	1.0	Rupert Murdoch (American businessman)	1.0	Peter Thiel (billionaire entrepreneur, venture capitalist)	1
10/18/2019	Scott McLean (journalist/podcaster)	2.0	Satoshi Nakamoto (developer of bitcoin)	2.0	Donald Trump (US President at the time)	2.0
11/15/2019	Satoshi Nakamoto (developer of bitcoin)	4.0	Brett Wilson (Canadian investment banker)	1.0	Tyler Mathisen (American journalist)	1.0
12/13/2019	John Green (author)	1.0	Jeff Bezos (billionaire CEO of Amazon)	1.0	NA	NA
01/10/2020	Matthew Rossi (Co-Founder Co-Founder Data Union DAO)	8.0	Henry Ford	3.0	Roger Ver (investor in Bitcoin)	1.0
02/07/2020	NA	NA	NA	NA	NA	NA
03/06/2020	Satoshi Nakamoto (developer of bitcoin)	1.0	Dave Perrill (CEO of Compute North)	1.0	Olga Kharif (Journalist)	1.0
04/03/2020	NA	NA	NA	NA	NA	NA
05/01/2020	Mati Greenspan (CEO & Founder, Quantum Economics)	4.0	Satoshi Nakamoto (developer of bitcoin)	3.0	Prince Harry (British royalty)	2.0
05/29/2020	Lawry Trevor (Co-Founder, HouseBit & President, Strathmere Assoc. Int. Ltd)	1.0	Dan Held (Director of Growth Marketing at Kraken)	1.0	NA	NA

**Table 5:** Person entities—Topic 7

Date	TOPIC 7					
	First Entity	Freq	Second Entity	Freq	Third Entity	Freq
05/01/2018	John McMahon (CEO of Prophecy Defi)	2.0	David Baddiel (English comedian)	2.0	Tom Lee (Managing Partner, Fundstrat Global Advisors)	1.0
05/29/2018	Mati Greenspan (CEO & Founder, Quantum Economics)	8.0	Lea Thompson (Girl Gone Crypto Founder)	3.0	Patrick Bryne (former CEO of Overstock)	1.0
07/27/2018	Tai Lopez (American businessman)	1.0	Sergey Nazarov (co-founder of Chainlink)	1.0	NA	NA
08/24/2018	Cody Wilson	2.0	NA	NA	NA	NA
09/21/2018	Satoshi Nakamoto (developer of bitcoin)	1.0	NA	NA	NA	NA
10/19/2018	Frank Pallone (US Congressman)	2.0	Richard Dennis (CEO at Temtum)	1.0	NA	NA
11/16/2018	Satoshi Nakamoto (developer of bitcoin)	2.0	Oleg Sharpaty (The Founder of W12)	1.0	NA	NA
12/14/2018	Lady Gaga (American singer)	1.0	Jeff Bezos (billionaire CEO of Amazon)	1.0	Bram Cohen (American computer programmer and bitcoin investor)	1.0
01/11/2019	Ethan Lou (Canadian journalist)	9.0	Dmitry Muraschchik (Community Manager for Mycelium)	1.0	NA	NA
02/08/2019	Alexandria Ocasio-Cortez (American politician)	3.0	Elon Musk (business magnate and investor)	2.0	Bernie Sanders (US politician)	2.0
03/08/2019	Roy Spencer (scientist)	1.0	Jing Liu (Chairman of ABCMint)	1.0	NA	NA
04/05/2019	Max Keiser (American broadcaster)	2.0	Xi Jinping (President of China)	1.0	NA	NA
05/03/2019	Elon Musk (business magnate and investor)	2.0	Vitalik Buterin (co-founder of Ethereum)	1.0	Teresa May (Former Britist Prime Minister)	1.0
05/31/2019	Peter McCormack (journalist/podcaster)	3.0	NA	NA	NA	NA
05/28/2019	Elon Musk (business magnate and investor)	20.0	Jeff Bezos (billionaire CEO of Amazon)	2.0	Donald Trump (US President at the time)	2.0
07/26/2019	Olivia Voz (American journalist)	1.0	NA	NA	NA	NA
08/23/2019	Vitalik Buterin (co-founder of Ethereum)	1.0	Tuur Demeester (Independent investor and commentator)	1.0	Robert F. Kennedy (Chairman of Childrens Health Defense)	1.0
09/20/2019	Jeff Bezos (billionaire CEO of Amazon)	1.0	NA	NA	NA	NA
10/18/2019	Donald Trump (US President at the time)	2.0	Tony Hawk (American Skateboarder)	1.0	Nancy Pelosi (US congresswoman)	1.0
11/15/2019	Tommy Robinson (activist)	1.0	Randy Brito (Founder of bitcoin Venezueala)	1.0	Frank Fannon (Managing Director, Fannon Global Advisors)	1.0
12/13/2019	Satoshi Nakamoto (developer of bitcoin)	1.0	NA	NA	NA	NA
01/10/2020	Jed McCaleb (co-founder and CTO of Stellar)	1.0	Brad Garlinghouse (CEO of Ripple, crypto solutions)	1.0	NA	NA
02/07/2020	Warren Buffett (American business magnate, investor)	2.0	Narendra Modi Prime Minister of India)	1.0	NA	NA
03/06/2020	Steve Grasso (CEO of Grasso Global Inc)	1.0	NA	NA	NA	NA
04/03/2020	Satoshi Nakamoto (developer of bitcoin)	2.0	Donald Trump (US President at the time)	1.0	NA	NA
05/01/2020	Satoshi Nakamoto (developer of bitcoin)	2.0	Victor Sauers (CEO at TKO Energy Capital)		Richard Dennis (CEO of Temtum)	1.0
05/29/2020	Elon Musk (business magnate and investor)	1.0	Elon Musk (business magnate and investor)	1.0	NA	NA

**Table 6:** Place entities

Date	TOPIC 3						TOPIC 4					
	First Entity	Freq	Second Entity	Freq	Third Entity	Freq	First Entity	Freq	Second Entity	Freq	Third Entity	Freq
05/01/2018	USA	4.0	Canada	1.0	Australia	1.0	Puerto Rico	2.0	NA	NA	NA	NA
05/29/2018	India	94.0	USA	2.0	Nigeria	2.0	USA	1.0	NA	NA	NA	NA
07/27/2018	India	28.0	USA	7.0	NA	NA	NA	NA	NA	NA	NA	NA
08/24/2018	USA	3.0	Ireland	2.0	Iceland	2.0	USA	3.0	NA	NA	NA	NA
09/21/2018	Switzerland	6.0	Uganda	1.0	USA	1.0	NA	NA	NA	NA	NA	NA
10/19/2018	China	3.0	Switzerland	2.0	Germany	2.0	NA	NA	NA	NA	NA	NA
11/16/2018	USA	2.0	NA	NA	NA	NA	NA	NA	NA	NA	NA	NA
12/14/2018	USA	2.0	China	1.0	NA	NA	NA	NA	NA	NA	NA	NA
01/11/2019	Canada	1.0	Paraguay	1.0	Austria	1.0	NA	NA	NA	NA	NA	NA
02/08/2019	USA	6.0	Canada	1.0	NA	NA	NA	NA	NA	NA	NA	NA
03/08/2019	USA	10.0	Singapore	5.0	Germany	4.0	NA	NA	NA	NA	NA	NA
04/05/2019	Italy	9.0	Spain	5.0	USA	3.0	NA	NA	NA	NA	NA	NA
05/03/2019	China	7.0	Italy	6.0	USA	6.0	USA	1.0	NA	NA	NA	NA
05/31/2019	USA	11.0	Australia	3.0	NA	NA	NA	NA	NA	NA	NA	NA
05/28/2019	USA	17.0	Switzerland	3.0	France	3.0	NA	NA	NA	NA	NA	NA
07/26/2019	Germany	3.0	Spain	1.0	NA	NA	NA	NA	NA	NA	NA	NA
08/23/2019	USA	2.0	Mexico	2.0	London	2.0	Thailand	1.0	NA	NA	NA	NA
09/20/2019	USA	1.0	Turkey	1.0	Spain	1.0	NA	NA	NA	NA	NA	NA
10/18/2019	USA	1.0	Ireland	1.0	Germany	1.0	Germany	1.0	NA	NA	NA	NA
11/15/2019	Russia	1.0	NA	NA	NA	NA	NA	NA	NA	NA	NA	NA
12/13/2019	USA	2.0	Italy	1.0	NA	NA	NA	NA	NA	NA	NA	NA
01/10/2020	USA	4.0	Turkey	1.0	NA	NA	NA	NA	NA	NA	NA	NA
02/07/2020	Italy	1.0	NA	NA	NA	NA	USA	1.0	NA	NA	NA	NA
03/06/2020	USA	3.0	NA	NA	NA	NA	Canada	1.0	USA	1.0	NA	NA
04/03/2020	USA	2.0	Guyana	1.0	Australia	1.0	NA	NA	NA	NA	NA	NA
05/01/2020	Thailand	2.0	NA	NA	NA	NA	NA	NA	NA	NA	NA	NA
05/29/2020	NA	NA	NA	NA	NA	NA	NA	NA	NA	NA	NA	NA

**Table 7:** Place entities (continued)

Date	TOPIC 6						TOPIC 7					
	First Entity	Freq	Second Entity	Freq	Third Entity	Freq	First Entity	Freq	Second Entity	Freq	Third Entity	Freq
6/1/2018	China	12.0	USA	8.0	Canada	5.0	USA	111.0	Switzerland	4.0	Puerto Rico	2.0
6/29/2018	Dubai	21.0	USA	34.0	NA	NA	USA	5.0	Paris	2.0	France	2.0
7/27/2018	USA	32.0	China	9.0	NA	NA	Canada	39.0	USA	2.0	France	2.0
8/24/2018	Iran	16.0	USA	19.0	NA	NA	USA	19.0	Venezuela	1.0	NA	NA
9/21/2018	China	74.0	Switzerland	52.0	USA	13.0	USA	4.0	Swiss Alps	4.0	Ontario	2.0
10/19/2018	Switzerland	29.0	China	23.0	USA	10.0	USA	5.0	Turkey	1.0	NA	NA
11/16/2018	USA	43.0	Iran	7.0	China	7.0	USA	12.0	China	5.0	Venezuela	1.0
12/14/2018	USA	40.0	China	7.0	Iran	3.0	China	18.0	USA	19.0	France	2.0
1/11/2019	USA	38.0	China	7.0	Spain	4.0	USA	12.0	China	2.0	NA	NA
2/8/2019	USA	20.0	China	6.0	Australia	3.0	USA	14.0	France	2.0	NA	NA
3/8/2019	USA	13.0	Venezuela	7.0	Hungary	4.0	Germany	6.0	USA	5.0	Canada	3.0
4/5/2019	China	38.0	Belarus	17.0	Iran	5.0	China	3.0	USA	2.0	Nigeria	2.0
5/3/2019	Canada	10.0	China	8.0	USA	7.0	China	3.0	USA	2.0	United Arab Emirates	1.0
5/31/2019	China	25.0	USA	22.0	New Zealand	9.0	USA	4.0	Venezuela	2.0	India	2.0
6/28/2019	Iran	35.0	USA	22.0	China	15.0	USA	4.0	India	3.0	Venezuela	2.0
7/26/2019	USA	16.0	China	5.0	Malaysia	3.0	USA	1.0	Jordan	1.0	UK	1.0
8/23/2019	Ukraine	5.0	USA	3.0	China	3.0	USA	4.0	China	2.0	Venezuela	1.0
9/20/2019	Belarus	12.0	China	6.0	USA	4.0	USA	3.0	India	2.0	China	2.0
10/18/2019	China	16.0	USA	10.0	Iceland	8.0	USA	6.0	China	2.0	NA	NA
11/15/2019	China	12.0	USA	3.0	Japan	2.0	Saudi Arabia	1.0	India	1.0	NA	NA
12/13/2019	Australia	26.0	USA	6.0	China	5.0	USA	3.0	Australia	1.0	Iran	1.0
1/10/2020	USA	10.0	China	5.0	NA	NA	Nigeria	1.0	Germany	1.0	France	1.0
2/7/2020	USA	6.0	China	3.0	UK		France	3.0	USA	2.0	UK	1.0
3/6/2020	USA	5.0	Russia	3.0	China	3.0	Lebanon	6.0	USA	10.0	NA	NA
4/3/2020	USA	11.0	Venezuela	2.0	NA	NA	China	7.0	USA	5.0	NA	NA
5/1/2020	China	40.0	UK	4.0	USA	3.0	France	55.0	Portugal	35.0	Finland	21.0
5/29/2020	Nigeria	1.0	Iran	1.0	NA	NA	France	11.0	Finland	8.0	Portugal	1.0

## Footnotes

1 The total value of all the coins that have been mined.

2 a shared database or ledger as discussed in Sect. 1
